# Supplementary material for: Electrochemically Oxidized Carbon Nanotube Sheets for High-Performance and Flexible-Film Supercapacitors
Source: Nanomaterials (Basel). 2023 Oct 23;13(20):2814. doi: 10.3390/nano13202814 (PMC10609474; doi:10.3390/nano13202814)
Supplement: Supplementary file 1 [file nanomaterials-13-02814-s001.zip › nanomaterials-2658119-supplementary.pdf]

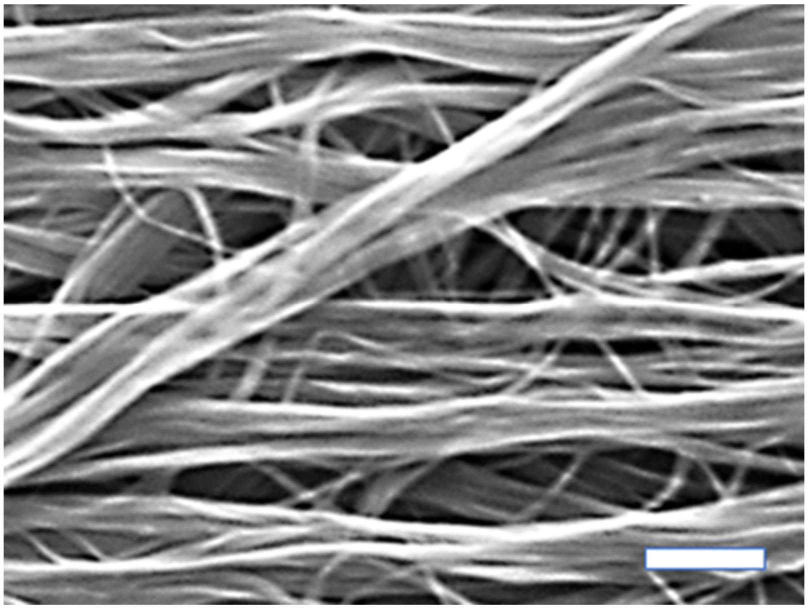

Figure S1. A scanning electron microscopy (SEM) image of carbon nanotube (CNT) sheets after electrochemical oxidation treatment (EOT) (scale bar = 200nm).

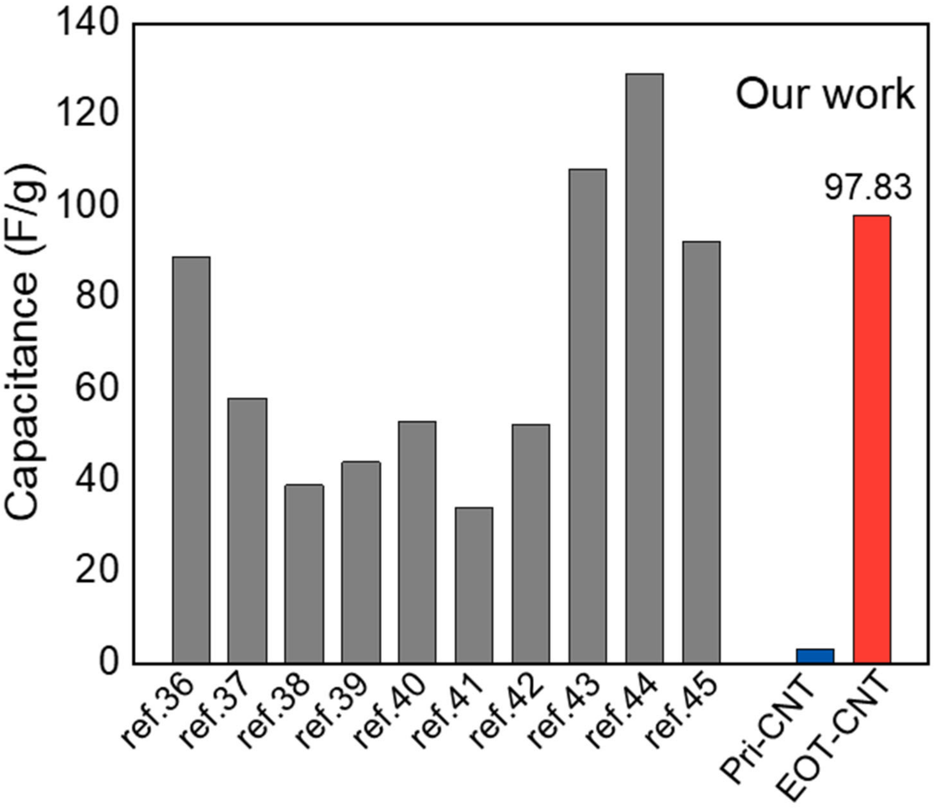

Figure S2. Capacitance comparison of the EOT CNT supercapacitor in the present work with previously reported CNT-based ones.

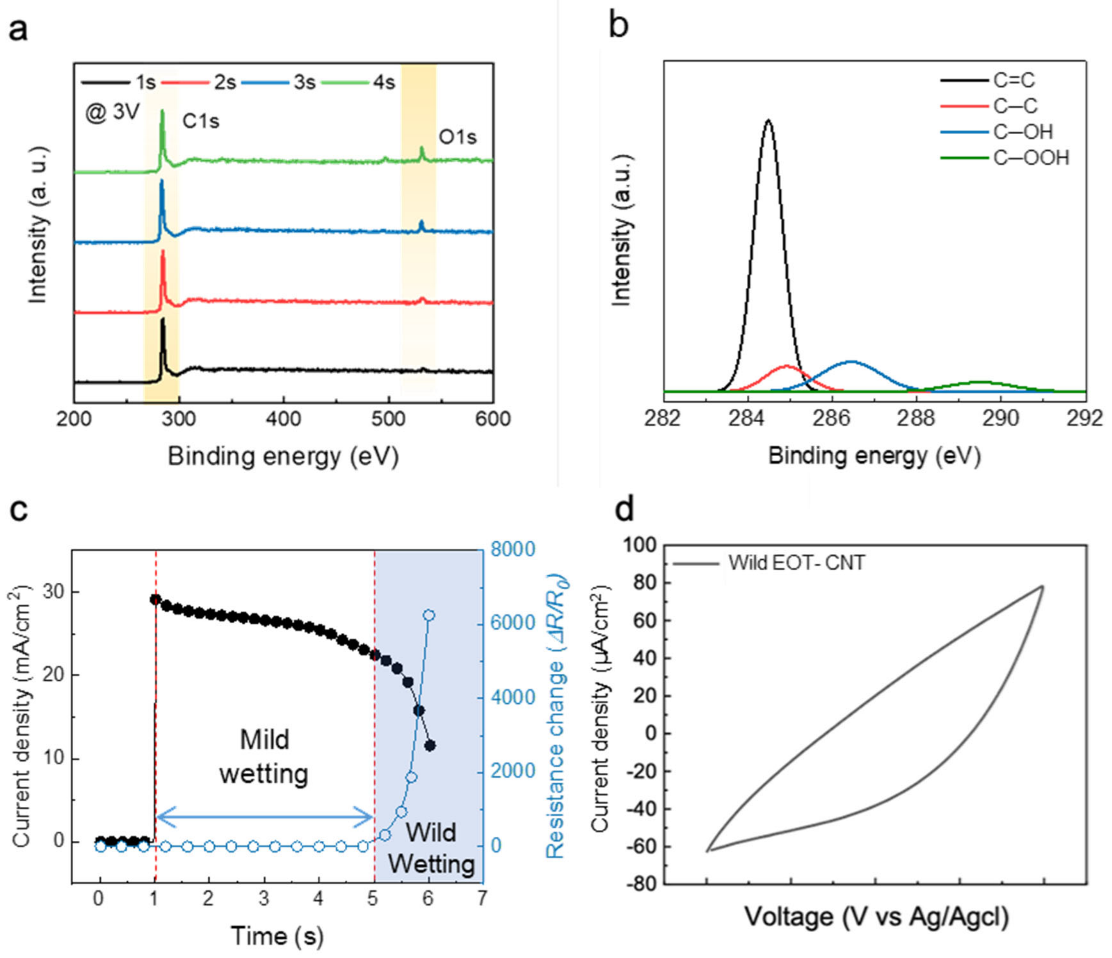

Figure S3. (**a**) An X-ray photoelectron spectroscopy (XPS) spectrum for EOT CNT sheet electrodes fabricated over various EOT times. (**b**) Deconvoluted C 1s XPS spectrum showing various functional groups. (**c**) Measured current density and resistance change of the EOT CNT working electrode versus EOT time (the mild and wild wetting regions are indicated). (**d**) Cyclic voltammetry (CV) curves of the wild-wetted CNT electrode.

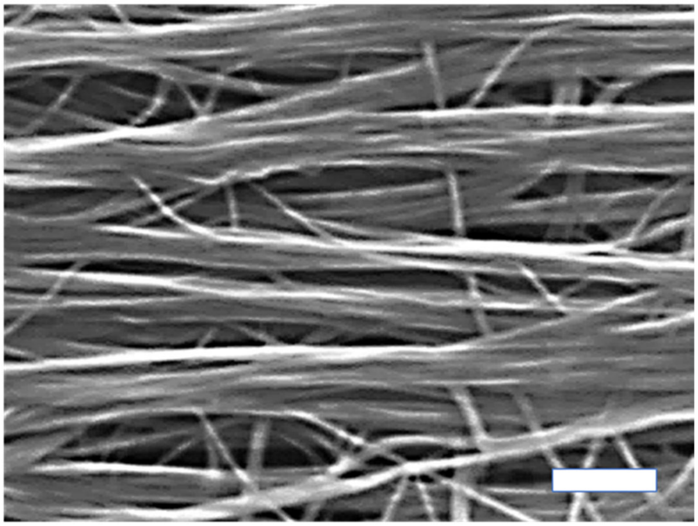

Figure S4. SEM images of the CNT working electrode after the 1000 bending cycle (scale bars = 200nm).
